# Supplementary material for: Exploring the Magnetic and Electrocatalytic Properties of Amorphous MnB Nanoflakes
Source: Nanomaterials (Basel). 2023 Jan 11;13(2):300. doi: 10.3390/nano13020300 (PMC9862160; doi:10.3390/nano13020300)
Supplement: Supplementary file 1 [file nanomaterials-13-00300-s001.zip › nanomaterials-2139021-supplementary.pdf]

Supplementary Materials

Exploring the magnetic and electrocatalytic properties of amorphous MnB nanoflakes

Boxiao Fu[1], Vasileios Tzitzios[2], Qiancheng Zhang[3], Brian Rodriguez[3], Michael Pissas[2], M. Veronica Sofianos[1,*]

[1]School of Chemical and Bioprocess Engineering, University College Dublin, Belfield, Dublin 4, Ireland; boxiao.fu@ucdconnect.ie (FB)
[2]Institute of Nanoscience and Nanotechnology, National Centre for Scientific Research "Demokritos", 15310 Athens, Greece; v.tzitzios@inn.demokritos.gr (VT); m.pissas@inn.demokritos.gr (MP)
[3] School of Physics, and Conway Institute of Biomolecular and Biomedical Research, University College Dublin, Dublin 4, Ireland; qiancheng.zhang@ucdconnect.ie (QZ); brian.rodriguez@ucd.ie (BR)
*Correspondence: mvsofianou@gmail.com

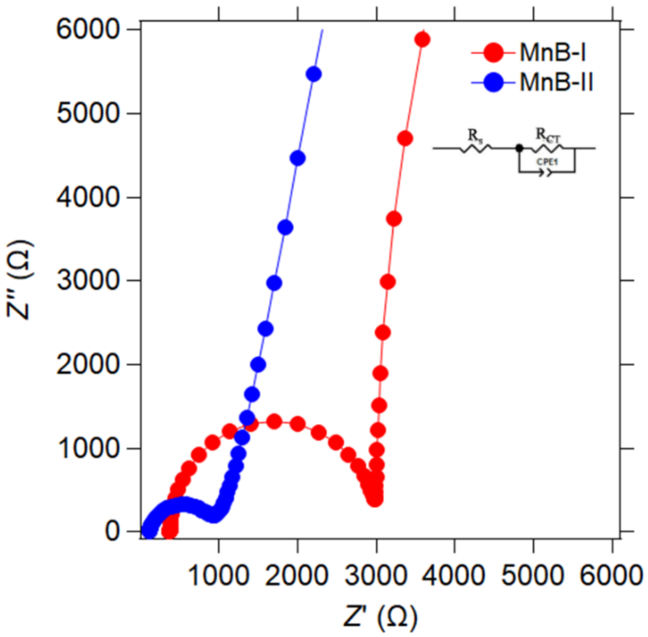

Figure S1. Nyquist plots obtained from EIS for the cobalt boride electrodes, and the inset shows the equivalent circuit.

Table S1. Representing the resistance values obtained by fitting the EIS data from Nyquist plots.

Catalysts	R_s (Ω)	R_{CT} (Ω)
MnB-I	337.3	3006.9
Mn-II	117.6	935.1

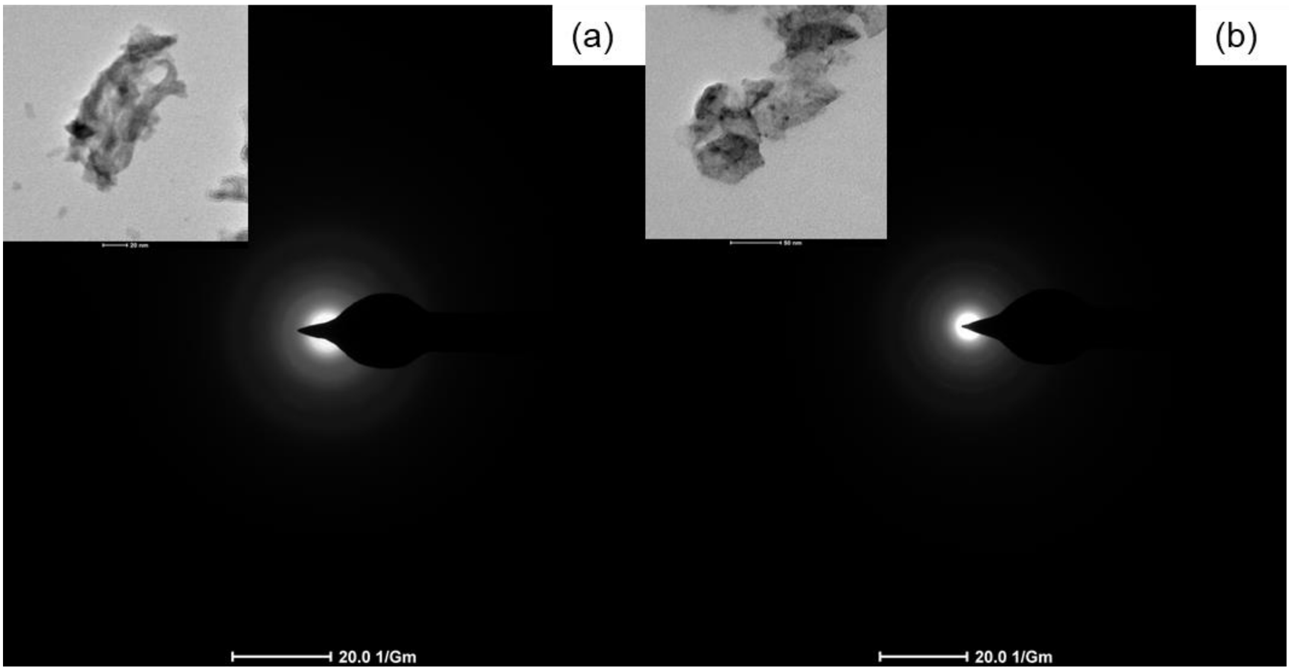

Figure S2: SAED pattern of (a) MnB-I and (b) MnB-II.
